# Factors associated with death in children with purpura fulminans: a French national prospective cohort study

**DOI:** 10.1186/s13054-021-03603-8

**Published:** 2021-05-28

**Authors:** Fouad Madhi, Naim Ouldali, Corinne Levy, Muhamed-Kheir Taha, Robert Cohen, The French Pediatric Meningitis Network, Abdelhadi, Aberrane, Abi Warde, Abou Tara, Abrudan, Adam, Adamon, Akitani, Alba-Sauviat, Al Chaar, Albertini, Aljazayri, Aljumaidi, Allali, Allia, Al Mazini, Amama, Amara, Amira, Amirault, Andriantahina, Ansoborlo, Arlet, Armengaud, Armouche, Arnault, Asensio, Aubert, Auburtin, Audry, Aujard, Autret, Azemar, Bachelier, Badran, Bajolle, Baleine, Bandin, Banegas, Banerjee, Banige, Barbier, Bardou, Baret, Barnaud, Baron-Joly, Barrans, Barraud, Barré, Barrey, Barsotti, Barthez, Bartizel, Baruteau, Basmaci, Bassil, Battaglini, Bayle, Bednarek, Belgaid, Belivier, Bellulo, Benabdelmalek, Benchekroun, Benezech, Bengrina, Benmahammed, Benmoulai, Benoist, Benoit, Bensaid, Benseddik, Benzaim, Berche, Berrahma, Beretta-Salaun, Bergounioux, Berrahma, Berterottiere, Berthier, Bertrou, Besson-Leaud, Biendo, Biessy, Billaud, Billion, Bina, Bineau, Biran, Bitar-Obeid, Blanc, Blasquez, Blondel, Blondin, Boileau, Boivin, Boize, Bolot, Bonacorsi, Boniface, Bongo Boina, Bonnin, Boquet, Bordes, Born, Bosdure, Bosi, Bost-Bru, Bouainane, Bouderlique, Boukezia, Boulard, Bour, Bourennane, Bourgeois-Nicolaos, Boussadia, Boussicault, Boutros, Boutte, Bouvet, Bouziges, Bovero, Boyer, Branca, Branger, Bresson, Breton, Breuil, Briand, Brieu, Brizard, Brocard, Brouard, Bruna, Brunel, Bruno, Bruyas, Bucher, Bucur, Buisson-Touati, Bulteau-Cowan, Cabaret, Cadot, Caillon, Callamand, Calvez, Cambonie, Campet, Canis, Canzi, Capdeville, Carbajal, Carbonnelle, Carre, Carre-Cavellier, Carrer, Carriere, Carroger, Cartier, Cartier-Riviere, Cascarigny, Castella, Castelnau, Cathenoz, Cattoen, Cattoir, Cau, Cavalier, Cecille, Chabrol, Chace, Chaix, Chale, Chalumeau, Chalvon Demersay, Chami, Chamouilli, Chamoux, Champion, Chandesris, Chantelat, Chantepie, Chaplain, Chappet, Charachon, Charbonneau, Chardon, Charras, Chenaud, Chenel, Cheron, Cheuret, Chevallier, Chevrel, Chomienne, Chrisment, Ciupek, Clair, Claris, Cloix, Coche, Coinde, Cointin, Colin Gorski, Collignon, Colombani, Combe, Constanty, Cordier, Cormier, Corniau, Cosson, Costa, Cottin, Coumenges, Coupe, Courcol, Courdavault, Courouble, Courtade, Craiu, Crepet, Croizé, Cuzzi, D’albignac, Dagorne, Dalmon, Daltroff, Danan, Danekova, Danjean-Deguin, Danjoux, Dao-Dubremptz, Daoud, Daoudi, Darai, Darras, Dassieu, Dauger, Debriel, Dechamps, Decobert, Decoster, Decousser, Deforche, Degas, Degrange, Delacour, Delamare, Delaporte, Delarbre, Delattre, Delavelle, Delbeke, Delesalle, Deligne, Delisle, Delvart, Delorme, Deluca, Demachy, Demarcq, Demarque, Demay-Legros, Demersay, Demonchy, De Barbentane, De Champs, De Decker, De Montclos, De Mongolfier, De Pontual, Denis, De Ricaud, Desbois, Desfrere, Desprez, Dessein, Dessioux, Desvigne, Deutscher, Devictor, Deville, Devouge, Dhaoui, Dieckmann, Dit Dinard, Djafari, Djaghri, Doit, Dolfi Fiette, Dolhem, Dommergues, Dorangeon, Doucet, Doucet-Populaire, Douchain, Dubois, Dubos, Dubourdieu, Dubus, Duchaine, Duchene, Ducrocq, Dufillot, Dufour, Dugain, Dumont, Dumoulard, Dupre, Duquesne, Durand, Duthilly, Dutron, Duval, Duval Arnould, Eb, Eicher, Eitenschenck, El Hamri, Elbez, Elharrif, Elias, Emond, Enchery, Enoch, Epaud, Escarguel, Estapa, Esteve, Estournet Mathiaud, Estrangin, Etienne, Evers, Evrard, Evreux, Eyer, Eyssette-Gayraud, Ezzedine, Fabbro, Faibis, Fargeot, Farges, Farhat, Farrugia, Fasquelle, Faul, Favaretto, Faye, Feldmann, Ferey, Ferroni, Fevre, Fieschi, Fiette, Fihman, Filippi, Fischbach, Flevin, Flipo, Flurin, Forget, Fortin, Fos, Foskett, Foucaud, Franc, Francois-Chervet, Francoise, Frey, Gagliardone, Gaillard, Gallet, Gallou, Ganivala, Garandeau, Garbarg-Chenon, Garcera, Garnier, Garrec, Gaschet, Gaschignard, Gaudelus, Gauduchon, Gaugler, Gavignet, Gbadamassi, Geffroy, Gendrel, Geniez, Georget, Geraudel, Gerony-Laffitte, Gilles, Gillet, Giorgi, Girard, Gire, Girier, Glastre, Glatz, Gleize, Goguelin, Goissen, Goldstein, Goudeau, Gougeon, Goumy, Gouraud, Gouriet, Goux, Graber, Graff, Grailles, Graine, Grancher, Grando, Granier, Granry, Greco, Gremeaux, Gremillet, Gressier, Grimprel, Grise, Grollmuss, Guerin, Guibert, Guichard, Guiet, Guigonis, Guilbert, Guillaume Baudet, Guillermet Fromentin, Guillois, Guillot, Guilluy, Guitton, Guyon, Haas, Hachani, Hachart, Hachem, Haegy-Doehring, Hage, Halfatuhi, Hallage, Hallalel, Halna, Halphen, Hamdad, Haouisee, Harchaoui, Hasselmann, Hautefort, Hedjem, Hees, Heidt, Hentgen, Heraud, Herve, Heurte, Heusse, Hevin Martin, Heyman, Hidri, Hochart, Hoffmann, Hombrouck-Alet, Honore, Horea, Hubert, Hudebine, Huet, Huguet, Huin, Hureaux, Huvenne, Ikounga, Issa-Brunet, Izopet, Jacob, Jalloul, Jan, Jaouen, Jarlier, Jarreau, Jaulhac, Javouhey, Jeannoel, Jeannot, Jehan, Jensen, Jeny, Jeudy, Jokic, Joly-Guillou, Joly-Sanchez, Joram, Jorion, Julienne, Jullian, Juvin, Kalach, Kalkas, Kayal, Kayemba, Khaled, Khalfi, Khazaal, Khorsi, Kitzis, Klein, Klink, Klosowski, Kone-Paut, Kouame, Kovacs, Kozisek, Kretz, Kucerova, Kuntzel, Labarthe, Labbe, Labenne, Laborie, Labrune, Lafargue, Lafendi, Lagier, Lagree, Lahrach, Laidj, Laisney, Lakhdari, Lamarcq, Lambert, Lamoureux, Landragin, Lanotte, Lapeyre, Larchet, Laugel, Launay, Lavigne, Layadi, Lazarro, Le Bail, Le Bideau, Le Coustumier, Le Galloudec, Le Luyer, Leblanc, Leboucher, Lebrun, Lecarpentier, Lecine, Leclerc, Leclercq, Lecomte, Ledru, Lefevre, Lefrand-Crepin, Legagneur, Legros, Leheup, Lehnert, Lehours, Lejri, Lelioux, Lelorier, Leluan, Lemarié, Lemble, Lemeland, Lemonnier, Lenclen, Leneveu, Leotard, Lepage, Leret, Lesage, Lescanne, Letellier, Leturdu, Levy, Lienhardt, Lorrot, Louf, Lounis, Loznewski, Lureau, M’bamba, Maakaroun-Vermesse, Macchi, Madhi, Mager, Magrhraoui, Mahisatra, Maisonneuve, Malbrunot, Malige, Mallet, Mammeri, Mammes, Mancini, Mandjee, Mangeol, Manin, Mansir, Manteau, Marani, Marchand, Marcou, Marguet, Mariette, Marin, Marmonier, Marmouset, Marret, Martha, Martin, Martinat, Martinez Ozenda, Martinot, Marty, Mathieu, Matray, Maugard, Maurage, Maurin, May, Mazataud, Mellier, Melon, Menager, Menget, Menouar, Menouni-Foray, Merlot, Mermet Jeanvoine, Meslin, Meunier, Meyer, Mezgueldi, Micaelo, Mignard, Mignot, Mikail, Milcent, Milh, Milleret-Proyart, Mitanchez, Mondaud, Monier, Monin, Montagnon, Moquet, Morales-Gineste, Moreigne, Morice, Moriette, Morin, Morisot, Morville, Motte, Mougin-Joubert, Moulene, Moulin, Mouly, Mounzer, Mselati, Muller, Muller, Murbach, Nacer, Naudion, Naudot, Navarro, Nelson, Nerl Schiavini, Nerome, Netter, Neuwirth, Nevine, Nguyen, Nicola, Nordmann, Noulard, Nsimba, Oprea, Orzechowski, Osman, Ould Hocine, Oules, Ozanne, Pages, Paget, Palette, Pancher, Pangon, Pannecouck, Paon, Parisi Duchene, Pateyron, Patoz, Paul, Paut, Pejoan, Pellerin, Pelloux, Penaud, Penel-Capelle, Peralta, Pere, Perez, Perrin, Perroud, Pesenti, Petit, Petitboulanger, Phan, Philippon, Picherot, Pierre, Pina, Pierrejean, Pietrement, Pigeon, Pina, Pinchi, Pindi, Pinquier, Pinturier, Pladys, Planchenault, Plassart, Plasse, Plouvier, Poilane, Poilly, Pollet, Pons, Popelard, Porcheret, Poulain, Poyard, Poynard, Pradeaux, Prere, Priqueler, Prudent, Py, Queinnec, Questiaux, Quinet, Raber, Rafin, Raignoux, Raoult, Rault, Raymond, Raynaud, Rebaud, Renault, Renaud Vialet, Rennes, Renolleau, Retali, Retornaz, Rey, Reynaud, Richardin, Rimet, Rimpici, Rio, Rivaux, Riviere, Roche, Rodiere, Rolland, Romain, Romeo, Rosellini, Rossignol, Roth, Rottman, Roubin, Roudaut, Roudière, Rougeoreille, Rougier, Roullaud, Rousseau, Roussellier, Roux, Roybet, Roze, Sachs, Sadik, Sadki, Saf, Said-Menthon, Sakr, Saliba, Salomon, Sanchez, Santerne, Sanyas, Sarlangue, Sarthou, Sartre, Saumureau, Saunier, Savoy, Scanvic, Scat, Schaefer, Schneider, Seaume, Sebag, Secher, See, Sevin, Sfez, Simonin, Siouala, Sirot, Sivadon-Tardy, Smati, Smets, Sommabere, Soto, Soustelle, Spicq, Stefaniuk, Stephan, Sweertvaegher, Tahiri, Tarral, Tchanga, Terki, Texier, Therond, Thevenot, Thibault, Thiriez, Thore, Thouvenin, Tiry, Tixier, Tommasi, Toubiana, Touroult-Jupin, Tourrand, Tran, Trioche, Troller, Tronc, Trouilloud, Tuypens, Tytgat, Ursulescu, Uzan, Vachee, Vaillant, Valade, Valayer, Valensi, Vallee, Vallet, Vandenesch, Van de Perre, Vanel Contamin, Varengue, Vasselon-Raina, Vaucel, Venot, Verdan, Verdet, Vergerond, Vergnaud, Vergne, Vernet-Garnier, Vervel, Viala, Vialette, Vic, Vignaud, Vigneron, Villeneuve, Violette, Vittecoq, Vodoff, Vogt, Vorlet, Vray, Vrillon, Vu Thien, Warin, Wasels, Weber, Weisse, Wemeau, Worcel, Yang Ting, Ygout, Youssef, Ythier, Zaoui, Zarzour, Zehani, Zelinsky-Gurung, Zenkhri, Zimmermann, Zitvogel, Zumbo

**Affiliations:** 1 Service de Pédiatrie Générale, Centre Hospitalier Intercommunal de Créteil, 40, avenue de Verdun, 94000 Créteil, France; 2 GPIP, Groupe de Pathologie Infectieuse Pédiatrique, Paris, France; 3 Université Paris Est, IMRB-GRC GEMINI, Créteil, France; 4 ACTIV, Association Clinique et Thérapeutique Infantile du Val-de-Marne, Saint Maur-des-Fossés, France; 5 Department of General Pediatrics, Pediatric Infectious Disease and Internal Medicine, Robert Debré University Hospital, Assistance Publique-Hôpitaux de Paris, Paris, France; 6 Université de Paris, INSERM UMR 1123, ECEVE, Paris, France; 7 Clinical Research Center (CRC), Centre Hospitalier Intercommunal de Créteil, Créteil, France; 8 Institut Pasteur, Unit of Invasive Bacterial Infections & National Reference Center for Meningococci, 75724 Paris, Cedex 15, France; 9 Unité Court Séjour, Petits nourrissons, Service de Néonatalogie, Centre Hospitalier Intercommunal de Créteil, Paris, France

To the editor,

As there is a high mortality rate and major risk of sequelae of purpura fulminans (PF), early identification of patients at highest risk of poor outcome is crucial to optimize the initial management. To date, only a few studies have suggested clinical or biological factors associated with an unfavorable evolution because of the rarity of PF in children. These studies were most often monocentric and retrospective [1, 2] or did not specifically study PF in children [3]. We analyzed the clinical and biological factors available at initial presentation associated with death in children with PF.

We conducted a national population-based prospective French cohort of pediatric PF over 15 years. From 2003 to 2017, we included all children < 15 years with PF with or without meningitis in one of the 227 participating centres. The methodology of this surveillance system was previously published [4]. The diagnosis of PF followed published recommendations [5] and remained unchanged over the study period. The main outcome was the case fatality rate before hospital discharge. We assessed demographic, clinical and biological factors, along with initial treatments used associated with death. For biological factors, we considered explorations performed at admission.

We included 1042 cases of PF: *N. meningitidis* was the most frequent bacterium isolated (910, 86.5%), including 581 with serogroup B (64.5%), 218 serogroup C (24.2%). Table 1 shows the demographic and clinical characteristics at admission and outcomes. Among 1042 patients with PF, 166 (16.1%; 95% CI [13.9; 18.5]) died during hospitalization. For the 876 survivors, 143 (16.3%; 95% CI [13.9; 18.9]) had acute complications or sequelae, including extensive skin necrosis (n = 56), neurological complications (n = 32), and limb amputation (n = 20). Table 2 summarizes the risk factors for death in children with PF. On multivariate analysis, death was significantly associated with age < 1 year and leucopenia < 5000/mm^3^; while leukocytosis > 10,000/mm^3^ was protective. Initial appropriate antibiotic treatment was not associated with death. Furthermore, death was faster with than without leucopenia (mean time to death 1.9 vs. 4.9 days, *p* = 0.013). Our multivariable model estimated that the probability of death could range from 3% for children without risk factors to 36% for children < 1 year old and with leucopenia < 5000/mm^3^.

**Table 1:** Demographic and clinical characteristics of purpura fulminans (PF) in children at admission, and intra-hospital outcomes, N = 1042

	**Total**
**Number of cases (%)**	1042
**Age (years), mean ± SD**	4.2 ± 4.2
**Median (n = 1042)**	2.5
**Sex (male) (n = 989)**	585 (59.1)
**Daycare modality (n = 852)**	
Home	372 (43.7)
Daycare centre	422 (49.5)
Child minder	58 (6.8)
**Underlying conditions (n = 1042)**	95 (9.1)
Prematurity (%)* (n = 587)	72 (6.9)
Cardiopathy (n = 846)	6 (0.7)
Acquired immunodeficiency (n = 838)	4 (0.5)
Congenital immunodeficiency (n = 974)	4 (0.4)
Recurrent meningitis (n = 960)	5 (0.5)
Asplenia (n = 919)	2 (0.2)
Cochlear implants (n = 712)	2 (0.3)
**Initial antibiotic therapy by 3GC**	1042 (100)
Antibiotic treatment within the 24 h before admission (%)* (n = 933)	425 (45.6)
**Clinical characteristics**	
Seizure before antibiotic therapy (%)* (n = 976)	78 (8)
Seizure after antibiotic therapy (%)* (n = 957)	58 (6.1)
Coma (%)* (n = 955)	294 (30.8)
Mechanical ventilation (%)* (n = 975)	433 (44.4)
Shock (%)* (n = 1002)	766 (76.5)
Complications (%)* (n = 1042)	143 (13.7)**
Neurological	32 (3.1)
Non-neurological (including imputations)	114 (10.9)
Limb amputation	20 (1.9)
**Death (%) (n = 1032)**	166 (16.1)
Time from admission to death (days), mean ± SD*	3.9 ± 11.3
**Median**	1

Data are n (%) unless indicated

^*^ % calculated on available data only

3GC: third generation cephalosporin

^**^ 3 patients had neurological and non-neurological complications

^***^ 1 patient had neurological and non-neurological complication

**Table 2:** Univariate and multivariate analysis of risk factors for death in children with purpura fulminans, N = 1042

Characteristics	Deaths n (%)	Univariate analysis		Multivariate analysis backward stepwise model	
		OR (95% CI)	p	OR (95% CI)	p
**Age (years)**					
0–1	56/228 (24.6)	3.4 [2.1; 5.4]	** < 0.001**	2.3 [1.4; 3.7]	**0.001**
1–2	37/193 (19.2)	2.5 [1.5; 4.1]	0.001	2.2 [1.3; 3.7]	**0.004**
2–4	28/212 (13.2)	1.6 [0.9; 2.7]	0.09	1.5 [0.9; 2.7]	0.124
> 4	32/365 (8.8)	1		1	
**Daycare modality**					
Child minder	8/57 (14.0)	1.3 [0.6; 3.0]	0.5		
Home	66/352 (18.8)	1.9 [1.2; 2.8]	**0.003**		
Daycare centre	45/410 (11.0)	1			
**Prematurity**					
No	64/495 (12.9)	1			
Yes	12/64 (18.8)	1.6 [0.8; 3.1]	0.2		
**Clinical profiles**					
PFMen +	82/629 (13.0)	1			
PFMen-	19/113 (16.8)	1.3 [0.8; 2.3]	0.3		
PFLP-	52/256 (20.3)	1.7 [1.2; 2.5]	**0.007**		
**Initial antibiotic therapy by 3GC**					
No	0				
Yes	166/1032 (16.1)	NA			
**Antibiotic treatment within the 24 h before admission**					
No	79/488 (16.2)				
Yes	45/407 (11.1)	0.6 [0.4; 1.0]	**0.028**		
**Blood culture**					
Positive	63/337 (18.7)	1.5 [1.0; 2.3]	0.051		
Negative	42/321 (13.1)	1			
**CRP level**					
< 100 mg/L	32/204 (15.7)	2.4 [1.2; 4.6]	**0.010**		
> 100 mg/L	14/194 (7.2)	1			
**Leukocyte count**					
< 5000/mm^3^	56/173 (32.4)	2.2 [1.3; 3.7]	**0.004**	2.1 [1.2; 3.6]	**0.006**
5000–10,000/mm^3^	26/144 (18.1)	1		1	
> 10,000/mm^3^	19/407 (4.7)	0.2 [0.1; 0.4]	** < 0.001**	0.3 [0.1; 0.5]	** < 0.001**
**Neutrophil count**					
< 1500/mm^3^	30/66 (45.5)	4.0 [2.2; 7.2]	** < 0.001**		
1500–10,000/mm^3^	43/251 (17.1)	1			
> 10,000/mm^3^	5/263 (1.9)	0.1 [0.0; 0.2]	** < 0.001**		

PFMen + : PF with meningitis; PFMen-: PF without meningitis; PFLP-: PF without lumbar puncture; LP: lumbar puncture; 3GC: third generation cephalosporin; CRP: C-reactive protein

To our knowledge, this 15-year population-based national study is the largest prospective study focusing on pediatric PF. We identified young age and leucopenia as independent risk factors for death in pediatric PF.

Younger age is a well-identified risk factor for poor outcomes in numerous pediatric invasive bacterial diseases and may be explained by the immaturity of the immune system [2, 3]. The findings regarding leukocytes may be less expected [2]. Several hypotheses should be considered. First, the lower leukocyte count in non-survivors suggests a shorter disease course and the completeness of the meningococcal infection. This suggestion may be supported by a shorter time from admission to death for children with leukopenia versus other children. Second, several immunological studies suggested that the development of neutropenia may indicate an exhaustion of bone-marrow progenitors, a maturation arrest in granulocytic lineage or an imbalance between extravasation and production [2, 6]. These mechanisms may also be associated with impaired neutrophil functions (chemotaxis, phagocytosis and production of reactive oxygen species), thus leading to poor outcomes during sepsis.

In conclusion, we identified several factors easily available at initial care independently associated with death. These findings may help physicians better appreciate the risk of short-term fatal evolution when managing PF in children. If these risk factors are not modifiable, their presence might lead clinicians to faster use aggressive therapies at initial care.

## Data Availability

The datasets used and/or analyzed during the current study are available from the corresponding author on reasonable request.
